# Estimation of Diagnosis and Treatment Costs of Non-Alcoholic Fatty Liver Disease: A Two-Year Observation

**DOI:** 10.5812/hepatmon.7382

**Published:** 2013-05-08

**Authors:** Mohammad Ebrahim Ghamar Chehreh, Mohsen Vahedi, Mohammad Amin Pourhoseingholi, Sara Ashtari, Hossein Khedmat, Mohsen Amin, Mohammad Reza Zali, Seyed Moayed Alavian

**Affiliations:** 1 Baqiyatallah Research Center for Gastroenterology and Liver Diseases, Baqiyatallah University of Medical Sciences, Tehran, IR Iran; 2 Department of Epidemiology and Biostatistics, Tehran University of Medical Sciences, Tehran, IR Iran; 3 Gastroenterology and Liver Disease Research Center, Shahid Beheshti University of Medical Science, Tehran, IR Iran

**Keywords:** Non-alcoholic Fatty Liver Disease, Costs and Cost Analysis, Delivery of Health Care, Utilization

## Abstract

**Background:**

There are insufficient data available on utilization and health care costs of non-alcoholic fatty liver disease. The cost data for different health conditions and services is a major gap in Iranian health system. So this study is the primary or first step towards filling this gap.

**Objectives:**

This study aims to estimate the diagnosis and treatment costs of Non-alcoholic Fatty Liver.

**Patients and Methods:**

This cross-sectional study was conducted on 528 subjects. The subjects had been diagnosed with non-alcoholic fatty liver. All the subjects had been referred to the Tehran Fatty Liver Clinic, a clinic of the Baqiyatallah Research Center for Gastroenterology and Liver Diseases, in 2009 and they had been observed for 2 years to determine the frequency of health care utilization (physician visit, laboratory tests, medication and cost of sonography). The costs of diagnosis and treatment for each person were estimated in Purchasing Power Parity dollars (PPP$).

**Results:**

The average total cost was 5,043 PPP$ per person in the 2 years of observation. Majority of these 528 patients (87.9%) had a BMI ≥ 25 (kg/m2). Also, 33.9% were diagnosed with comorbid diseases such as Diabetes Mellitus (DM), Coronary Artery Disease (CAD), hypertension (HTN) and hypothyroidism (HYPO).

**Conclusions:**

The results confirmed that the total costs for non-alcoholic fatty liver among the Iranian adult urban population alone exceeded 1 billion PPP$ per year. These costs can be saved or reduced by effective disease management and early prevention.

## 1. Background

Non-Alcoholic Fatty Liver Disease (NAFLD) is increasingly becoming the common cause of chronic liver diseases. One type of progressive NAFLD is known as Non-Alcoholic Steato-Hepatitis (NASH), which can lead to cirrhosis, hepatocellular carcinoma (HCC), liver dysfunction and ultimately death (1, 2). Nonalcoholic Fatty Liver Disease (NAFLD) encompasses a wide spectrum of changes; simple steatosis refers to > 5% of hepatic steatosis in the absence of significant inflammation and hepatocellular damage whereas NASH demonstrates inflammation and hepatocellular damage and sometimes fibrosis (3). Progressive NAFLD may result in liver cirrhosis, which may be complicated by HCC and liver failure. About 5% of patients with NAFLD develop cirrhosis over an average seven-year period with 1.7% mortality rate from complications of liver cirrhosis (4). Simple steatosis is comparatively benign with a 0-4% risk of cirrhosis development over a one to two decade period (5-7). In contrast, 5-8% of patients with NASH may develop cirrhosis over approximately five years (8-10). NAFLD is associated with several atherosclerotic risk factors such as hypertension, diabetes, and hypertriglyceridemia (11, 12). NAFLD is considered to be the hepatic manifestation of metabolic syndrome, and the links between obesity, diabetes, cardiovascular disease and NAFLD are likely to reflect shared pathogenetic factors. Roughly 90% of NAFLD cases have at least one characteristic feature of the metabolic syndrome, and one third of them fulfill the complete diagnostic criteria (13, 14). Most of patients with NAFLD are obese and suffer metabolic syndrome (15). NAFLD can impose enormous costs both on the patients and health and treatment system. Thus, there is no doubt that the availability of cost data for different health conditions and services is a major gap in the Iranian health system. Knowledge about the costs of an illness can help policy makers decide which diseases need to be addressed first by health care and prevention policies. Despite this, there are insufficient data available on the costs of NAFLD. Here, we have tried to fill this gap by conducting this study.

## 2. Objectives

Obesity and metabolic syndrome, the most predictive factors of fatty liver disease, are increasing in Iran; therefore, the prevalence of NAFLD/NASH and related complications are also expected to increase in the future (16). Accordingly, it is reasonable to assume that NAFLD progresses to more serious diseases, which result in higher utilization and cost of medical services. The present study aims to determine the NAFLD-related costs of diagnosis and treatment among NAFLD patients.

## 3. Patients and Methods

### 3.1. Patient Selection

For this cross-sectional study, 528 patients with NAFLD were selected from patients' referred to the Tehran Fatty Liver Clinic, a private clinic of the Baqiyatallah Research Center for Gastroenterology and Liver Diseases during 2009. The patient selection criteria were based on:

No history of alcohol consumption

No history of drug consumption (retrovirals, anti-seizures or statin use)

No history of chronic liver disease

Had regular visits for check ups

Had laboratory tests done regularly

Passed and completed a two year treatment period

The inclusion criteria were the presence of NAFLD as suggested by ultrasonography and rule out of alcohol or drug consumption and liver disease. The ultrasonography criterion was a hyper echoic texture or a bright liver because of diffuse fatty infiltration. Sensitivity and specificity for the ultrasound were 85 and 94 percent, respectively. The procedure for the diagnosis of NAFLD was as follows:

Evaluation of the serum aminotransferase (ALT, AST)

Evaluation of BMI > 25 (kg/m2); BMI over 25 is for overweight and obese people (17)

Evaluation of some anthropometric measurement criteria including body weight and height as well as the size of the wrist

Evaluation of the metabolic syndrome such as Diabetes Mellitus (DM), Coronary Artery Disease (CAD), hypertension (HTN) and hypothyroidism (HYPO)

And finally performance of ultrasound scan to identify NAFLD

In addition, some other variables including age, gender, behavioral risk factors like physical activity were also taken into consideration.

### 3.2. Cost Analysis

In this cross-sectional study, the diagnosis and treatment costs of NAFLD were estimated. The factors used for cost estimation included frequency of health resource utilization and unitary costs. Health resource utilization included such categories as physician visits (Patients were followed every 3-4 months by repeated laboratory studies based on primary laboratory disorders), laboratory tests, medication and cost of ultrasonography. The unit cost of different health resources including physician (GP/specialist) visits, laboratory tests, medication fees and the costs of ultrasonography were calculated based on the price lists approved in 2009-2011 by the Iranian Cabinet for the Public and Private Health Centers (18). The price of drugs was retrieved from the drug list of Food and Drug Office of Iranian Ministry of Health and Medical Education in 2009-2011 (19). Therefore, the unit cost of different health resources for each patient was calculated separately, based on their price during different years (according to the time the treatment had taken place). Total costs were the sum of all categories (physician visit, laboratory tests, medication and the costs of ultrasonography). Purchasing Power Parity Dollar (PPP$) was used in order to make inter-country comparisons. PPP$ is an economic technique used when attempting to determine the relative values of two currencies. It is useful because the amount of goods a currency can purchase within two nations often varies drastically; it depends on availability of goods, demand for the goods, and a number of other difficult-to-determine factors. According to the reports released by the Iranian Central Bank and World Bank Organization in 2010 (20); One PPP$ was estimated around 3894 in 2009, 4210 in 2010 and 4503 in 2011. This was then used to convert costs from Iranian Rials to PPP$. Using PPP$ is preferred to the US $, based on usual exchange rates, and make cross-country comparison of the costs more reliable.

### 3.3. Statistical Analysis

Descriptive statistics and frequency distribution such as mean, standard deviation and percentage were employed. T-test and one-way ANOVA was used to test the differences between means of NAFLD-related costs. All statistical analyses were carried out using SPSS, version 17.0 (SPSS Inc., Chicago. IL., USA). P < 0.05 was considered as statistically significant.

## 4. Results

In total, 528 patients with NAFLD were enrolled in this study out of which 320 patients (60.6%) were male and 208 (39.4%) were female. The mean age was 45.9 ± 10.8 (± standard deviation) years. The majority of them, 464 patients (87.9%), had a BMI ≥ 25 (Kg/m2). Also 179 individuals (33.9%) were diagnosed with comorbid diseases including DM, CAD, HTN and HYPO (Table 1).

**Table 1.** Demographic and Clinical Data of 528 Patients With NAFLD

	Male	Female	Total
**Age Group, No. (%)**			
≤ 50	235 (73.4)	115 (55.3)	350 (66.3)
> 50	85 (26.6)	93 (44.7)	178 (33.7)
**BMI ^a^, No. (%)**			
18-25	44 (13.8)	20 (9.6)	64 (12.1)
25-30	182 (56.9)	69 (33.2)	251 (47.5)
> 30	94 (29.3)	119 (57.2)	213 (40.4)
**DM, No. (%)**			
No	293 (91.6)	178 (85.6)	471 (89.2)
Yes	27 (8.4)	30 (14.4)	57 (10.8)
**CAD, No. (%)**			
No	305 (95.3)	192 (92.3)	497 (94.1)
Yes	15(4.7)	16 (7.7)	31 (5.9)
**HTN, No. (%)**			
No	267 (83.4)	159 (76.4)	426 (80.7)
Yes	53 (16.6)	49 (23.6)	102 (19.3)
**HYPO, No. (%)**			
No	310 (96.9)	183 (88.0)	493 (93.4)
Yes	10 (3.1)	25 (12.0)	35 (6.6)

^a^Abbreviations: BMI, body mass index; DM, diabetes mellitus; CAD, coronary artery disease; HTN, hypertension; HYPO, hypothyroidism

Total costs of NAFLD patients with and without comorbid diseases, and also with BMIs over 25 (over-weight and obese people) have been shown in Table 2. The Average cost for NAFLD patients without disease was 5123 PPP$ and the average cost for NAFLD patients with BMI > 25 was 5188 PPP$. The average cost of NAFLD patients with a metabolic syndrome was 4733 PPP$. Among the NAFLD patients with HTN the average costs were higher than the other patients.

**Table 2.** NAFLD-Related Costs in Patients With and Without Comorbid Diseases and BMI ≥ 25 (Costs Are Expressed in PPP$)

Comorbid Diseases	C.Dr^a^	C.M	C.T	C.S	Total Cost
**Without Diseases**	469	3548	1491	138	5123
**DM**	454	2415	1458	149	4222
**CAD**	526	2880	1502	129	4943
**HTN**	497	3227	1553	137	5126
**HYPO**	472	2933	1639	120	4643
**BMI ≥ 25**	482	3443	1531	138	5188
**Total Cost**	473	3348	1514	138	5043

^a^Abbreviation: CAD, coronary artery diseases; C.Dr, cost of physician visits; C.M, cost of medication; C.S, cost of sonography; C.T, cost of laboratory tests; DM, diabetes mellitus; HTN, hypertension; HYPO, hypothyroidism

Although the results indicated that the charges were higher in overweight and obese patients, yet these differences did not reach statistical significance (5.188 PPP$ vs. 4,005 PPP$; P = 0.061). This table also indicates no relationship between comorbid diseases and health care utilization and NAFLD-related costs (P = 0.649).

## 5. Discussion

In this study, data was collected from NAFLD patients’ files from a Tehran fatty liver private clinic from which costs of physician visit, laboratory tests, medication and ultrasonography was extracted from the price lists approved by the government and/or food and drug organization. It is worth noting that in estimating the unit costs of each health service, the minimum applicable cost was taken into account. This might have caused these estimated costs to be slightly lower than the actual costs. Although the charges were higher in overweight and obese patients, these differences did not reach statistical significant. One might assume that changes in patients' lifestyles, after the diagnosis of NAFLD, might have affected the results. The reason is that overweight and obese people, in this study, were first recommended to change their lifestyles by physicians. Also, the costs of the prescribed drugs, such as Orlistat, which reduce fat absorption and promote weight loss are much lower in comparison with the costs of other drugs in other countries (21, 22). In addition, perhaps another reason for the higher costs associated with overweight and obese patients is that the majority of the patients that participated in this study (87.8%) were overweight and obese. Also among NAFLD patients with comorbid disease, patients with HTN had higher costs (5,126 PPP$) than the other patients with comorbid disease, one might assume that the frequency of NAFLD patients with HTN (19.3%) were higher than the others. In the two-year observation, the total costs including costs of physician visits, laboratory tests, ultra songoraphy and medication totaled 5,043 PPP$ per person. Therefore, the diagnosis and treatment costs per person per year was estimated to be 2521 PPP$. Based on the available information on the costs of each NAFLD patient per year and the results of some population-based studies already performed in the country on prevalence of NAFLD, a rough estimate of yearly costs of diagnosis and treatment of NAFLD can be obtained. The study of Pourshams et al on healthy Iranian blood donors showed that the prevalence of NAFLD was 2.35% (23). Jamali et al reported a NAFLD prevalence of 2.04 percent for those over 18 years in Golestan provience of Iran. Also, the prevalence of NAFLD/NASH was reported to be about 2.9% among 18-year-old individuals in three geographically distinct provinces in Iran (Tehran, Golestan and Hormozgan), as reported by Sohrabpour et al. (2). Since, all patients who participated in this study were from urban areas, it can be assumed that the estimation for NAFLD costs per person only applies to urban adult population of Iran. According to the latest report published by the Statistical Center of Iran in 2006 (24), urban adult population was 17218066 million. If we estimate the prevalence of NAFLD from the studies, we may assume that NAFLD prevalence ranges from 2.04% to 2.9% (average: 2.4%) among adult urban population. Based on these findings, we reach a total cost estimation of 1 billion PPP$ per year. This amount only covers the costs of adults (18 and over) living in urban areas. This study also had some limitations. First, this study is limited to short-term follow-ups of two years. In order to consider the status of health care services, the observation of NAFLD progression and treatment may require longer follow-up periods and this is a subject to be studied for future research. Second, in order to calculate the estimated costs of treatment, the minimum cost has been considered for all health care services. This might have caused an underestimation of costs. Third, this study only measured costs of diagnosis and treatment of NAFLD; and the other inpatient and indirect costs, such as hospitalization, missed hours from work and school as well as transportation costs have not been taken into account. Despite the limitations of this study, the results are valuable in that they show the importance of NAFLD as a predictor of health care use and costs in an urban adult population. In conclusion, this study suggests that NAFLD seems to put a heavy burden on the economy of the country. However, the study limitation, as stated previously, must be considered; future studies should focus on longer follow-ups and include a wider spectrum of health care cost estimates, such as hospitalization, productivity loss, transportation, time spent by patients seeking care, costs incurred by caregivers and intangible costs such as emotional anxiety, fear, pain, suffering and stigmatization for realistic and precise estimations.
